# Modeling the variations in pediatric respiratory syncytial virus seasonal epidemics

**DOI:** 10.1186/1471-2334-11-105

**Published:** 2011-04-21

**Authors:** Molly Leecaster, Per Gesteland, Tom Greene, Nephi Walton, Adi Gundlapalli, Robert Rolfs, Carrie Byington, Matthew Samore

**Affiliations:** 1 Division of Epidemiology, University of Utah School of Medicine, Salt Lake City, USA; 2 Department of Pediatrics, University of Utah School of Medicine, Salt Lake City, USA; 3 Division of Disease Control and Prevention, Utah Department of Health, Salt Lake City, USA

## Abstract

**Background:**

Seasonal respiratory syncytial virus (RSV) epidemics occur annually in temperate climates and result in significant pediatric morbidity and increased health care costs. Although RSV epidemics generally occur between October and April, the size and timing vary across epidemic seasons and are difficult to predict accurately. Prediction of epidemic characteristics would support management of resources and treatment.

**Methods:**

The goals of this research were to examine the empirical relationships among early exponential growth rate, total epidemic size, and timing, and the utility of specific parameters in compartmental models of transmission in accounting for variation among seasonal RSV epidemic curves. RSV testing data from Primary Children's Medical Center were collected on children under two years of age (July 2001-June 2008). Simple linear regression was used explore the relationship between three epidemic characteristics (final epidemic size, days to peak, and epidemic length) and exponential growth calculated from four weeks of daily case data. A compartmental model of transmission was fit to the data and parameter estimated used to help describe the variation among seasonal RSV epidemic curves.

**Results:**

The regression results indicated that exponential growth was correlated to epidemic characteristics. The transmission modeling results indicated that start time for the epidemic and the transmission parameter co-varied with the epidemic season.

**Conclusions:**

The conclusions were that exponential growth was somewhat empirically related to seasonal epidemic characteristics and that variation in epidemic start date as well as the transmission parameter over epidemic years could explain variation in seasonal epidemic size. These relationships are useful for public health, health care providers, and infectious disease researchers.

## Background

Respiratory syncytial virus (RSV) has long been recognized as a substantial public health threat [1] with annual epidemics exacting an enormous toll on vulnerable populations and health care delivery systems. RSV is associated with substantial morbidity in children in both the hospitalized and outpatient setting [2-5]. In addition to the toll on the health of the population, this disease imposes a large burden on the health care system in terms of human and material resources. Although no RSV vaccine exists, infants and children with risk factors for severe RSV infection (eg, lung disease or prematurity) can receive monthly doses of palivizumab, a humanized murine anti-RSV monoclonal antibody, during the RSV season. Palivizumab treatment is extremely costly; the cost-effectiveness of this therapy could be improved if treatment is given only during times of high RSV activity. Treatment of vulnerable individuals also improves overall health in the population.

Prediction of seasonal epidemic characteristics including times of high activity and total size would support efficient management of resources and delivery of palivizumab. Health care facilities could forecast requirements for beds, staffing, testing, treatment, and other resources needed to care for sick children. For greatest effectiveness, these predictions should be made early in the RSV season; the authors, including public health practitioners and physicians, hold the expert opinion that these predictions would be useful within the first month of the observed start of the RSV seasonal epidemic.

**Figure 1:**
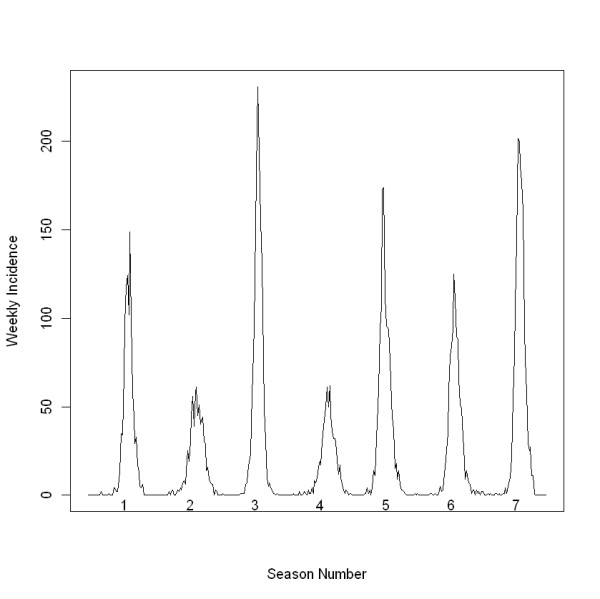
**Weekly observed RSV cases**. Weekly observed RSV cases for 7 epidemic years. Data collected from Primary Children's Medical Center in Salt Lake City from July 2001 through June 2008.

In some regions, total epidemic size generally follows a biennial cycle from year to year with smaller epidemic seasons followed by larger epidemic seasons [6]. This cycle is currently used to gauge upcoming RSV seasonal epidemic size based on total size of the previous epidemic season. The Centers for Disease Control and Prevention (CDC) researchers using the National Respiratory and Enteric Virus Surveillance System found that the prior epidemic season's data were a relatively imprecise predictor of the epidemic season onset in a given community and that timing of the RSV epidemic season may vary substantially in the same year among communities in close proximity [7]. One goal of this research was to explore year-to-year variation in epidemic seasons using local data. The biennial variation in our seasonal epidemic data was seen in the early exponential growth rates (slope of the cumulative case curves, Figure 1) as well as total epidemic size. We explored the relationship between exponential growth of RSV epidemics and the seasonal epidemic characteristics of total epidemic size, days to peak, and epidemic length to assess predictions made early in the epidemic season.

Knowledge about viral transmission characteristics and the data derived from surveillance systems can be used to inform novel approaches for estimating characteristics of RSV epidemics through the application of methods rooted in epidemiological models of infectious disease transmission [8,9]. These methods are being increasingly applied to emerging threats like SARS [10-12] and pandemic influenza, but their application to routine epidemics of common respiratory viruses like seasonal influenza and RSV has only begun to be explored. Weber *et al*. [8] model RSV transmission to examine how climate and social factors influence transmission in a population. They consider compartmental models using Susceptible-Infected-Recovered-Susceptible (SIRS) with additions to include latency and stages of susceptibility. They find no single best model for RSV epidemics; many "competing" models fit the observed data well. We further explored the variation in seasonal epidemics using compartmental models. The variation in exponential growth could potentially be related to variation in transmission rates, epidemic start dates, or proportions susceptible as well as a host of other factors.

The second goal of this research was to evaluate the ability of a compartmental model based on epidemiologic principles to fit observed data from a series of epidemics and examine the extent to which seasonal variations in epidemics can be accounted for by variation in specific model parameters.

For these analyses, we used daily laboratory data from the major pediatric health care facility in Utah where routine viral testing is a fixture of standard clinical care for children presenting to regional emergency departments. The utility of the data from these surveillance systems for relating final epidemic size and modeling the epidemic curve has not been fully evaluated. We investigated the estimation of seasonal epidemic characteristics using regression of exponential growth across seven epidemic seasons. We also modified the model of Weber *et al*. to explore the model fits and estimates of epidemic size using variation of parameters within a Susceptible-Exposed-Infected-Infected/Detected-Recovered (SEIDR) model.

## Methods

### Data

Primary Children's Medical Center (PCMC) is a 250-bed children's hospital that serves both as a community pediatric hospital for Salt Lake County, Utah (2008 population 1 million [13]), and as a tertiary referral center for five states in the Intermountain West (Utah, Idaho, Wyoming, Nevada, and Montana, total 2008 population 8.36 million [14]). Eighty percent of pediatric hospital admissions occurring in Salt Lake County and 73% occurring in the state of Utah are at PCMC.

During the study period, July 2001 through June 2008, direct respiratory sampling (mainly saline-assisted nasopharyngeal aspiration) for respiratory viral testing was performed for about 70% of children evaluated in the PCMC emergency department for respiratory complaints (unpublished data) and was required for all hospitalized children with respiratory symptoms (eg, upper or lower respiratory tract infection, bronchiolitis, asthma, or bacterial or viral pneumonia). In addition, respiratory viral testing was recommended for all febrile infants one to 90 days of age. Test results were used to inform patient cohorting and isolation procedures and to assist with medical management. All samples were initially tested by direct fluorescent antibody staining (DFA). DFA testing was performed three to five times daily depending on the season, with a mean turnaround time of four hours. For all DFA negative specimens, multiplex polymerase chain reaction (PCR) or viral culture was performed.

The data included in our analyses were all positive test results from the above sampling protocols from any of the testing methods during the study period. The practice of testing and test methods did not change appreciably during the study period (unpublished data on percentage of children tested and methods used). The data were used as daily counts by age group, under two and over two years old.

The RSV epidemic year was defined to be from July 1 of one year through June 30 of the following year. This time period was chosen to place the beginning date close to the middle of the inter-epidemic period, approximately six months from the average historical peak of the seasonal epidemic.

This study was reviewed by the Institutional Review Boards of Intermountain Healthcare and the University of Utah and determined by both organizations to be exempt.

### Regression analysis

Regression analysis was used to explore the relationship between the initial exponential growth rate and the epidemic season characteristics of size, days to peak, and length using the seven epidemic seasons of RSV data from PCMC. The exponential growth rate, λ_t0, t1_, for time interval t_0 _to t_1 _was calculated as , where  denotes the cumulative number of cases at time *t_i_, i = 0,1*. The exponential growth rate was calculated at four weeks to assess regression predictions made early in the season. For comparison, exponential growth rate was also calculated at weeks one through six. The total epidemic size was the sum of cases over the epidemic year, including sporadic inter-epidemic cases. An observable seasonal epidemic start date of t_0 _was defined as the start of the first week of the epidemic year with at least five confirmed RSV cases. This was the definition used by the hospital epidemiologists at PCMC to declare the start of RSV outbreaks during the study period. The term seasonal epidemic refers to the period from the epidemic start date until the epidemic end date, defined as the end of the last week of the epidemic year with at least five confirmed RSV cases. The number of days until the peak for the epidemic seasons was calculated as the midpoint day of the largest seven-day moving average window minus the epidemic season start day. The length of the epidemic season was calculated as the epidemic season end day minus the epidemic season start day.

Relationships between the initial exponential growth rate and seasonal epidemic characteristics were described using the Pearson correlation coefficient and assessed using standard regression statistics. The fits of the regression models were assessed using the percent error of the model fits from the observed values. To combine across seasons, the absolute values of the percent errors were averaged providing the mean absolute percent error for the model.

### SEIDR model

We modeled the observed RSV cases using an extension of the SIR model that included individuals (c for children and a for adults) that were susceptible (S^c ^and S^a^), exposed (E^c ^and E^a^), infectious(I^c ^and I^a^), infectious and subsequently detected children (D), and recovered combined across children and adults (R). This SEIDR model was applied to a series of seven epidemic years. The population was split into children less than two years old (children) and those older than two (adults). It has been shown that the initial RSV infection is the most severe and occurs in almost every child in their first two years of life. Transmission is modeled as a function of time using a cosine function to mirror the cyclic nature of epidemics [8]. There is an offset to this cycle (α), which we estimate along with transmission parameter (β). Births and deaths (μ) are accounted for in the susceptible class only. Achievement of age two is accounted for in all age-separated classes (η). Assumptions of simple compartmental models that we made were as presented in Koopman [15].

**Figure 2:**
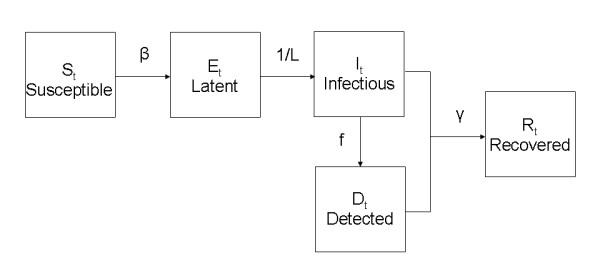
**Schematic representation of the flow through model compartments**.

Our SEIDR transmission model (Figure 2) was defined using the following system of non-linear differential equations:

Here β was the transmission parameter, *L *the latency period, *f *the under-two detection fraction, and γ the recovery parameter. All parameters are presented in the next subsection with descriptions, ranges, and reference values from the literature. Solution to the set of differential equations is addressed below.

### Model parameters

To fit the SEIDR model to the empiric epidemic data, three parameters-latency period, birth and death rate, and recovery period-were specified based on the literature. Three parameters associated with variation across epidemic years were estimated: 1) the temporal offset of the epidemic cycle (α), 2) detection fraction (f), and 3) transmission parameter (β). Different models were specified to explore the effect of these three parameters. All combinations of these were considered: models with one parameter allowed to vary across seasons, models with two parameters allowed to vary across seasons, and a model with all parameters allowed to vary across seasons.

Each parameter is described below.

#### Birth and death rate (μ)

The number of daily births and deaths were entered in the model based on census data for Salt Lake County.

#### Aging rate (η)

It was assumed that 1/365^th ^of the children in each age-separated compartment reached the age of two each day.

#### Detection fraction (f)

The detection fraction parameter reflected the fraction of the RSV epidemic in children under two years old that was captured in our data set. The detection fraction parameter was estimated as a constant parameter across years and also allowed to vary by epidemic year.

#### Latency period (L)

The latency period is the time between exposure resulting in transmission and time of infectiousness. The latency period was specified using the median value from Crowcroft [16], five days.

#### Transmission parameter (β)

The transmission parameter determined the rate of transmission from contacts between infectious and susceptible individuals. We assumed a homogeneous, uniformly mixing population. The transmission parameter was estimated as a constant parameter across years and also allowed to vary by epidemic year.

#### Recovery parameter (γ)

The recovery parameter specifies the time from infectiousness to recovery. This was specified as 0.1, which translates to a ten-day recovery period, following the work by Weber [8] and in the range of one to 21 reported by Hall [17].

#### Epidemic cycle offset (α)

The final model parameter was the offset of the annual epidemic cycle. A regular annual cycle is thought to vary due to weather and climate conditions. The SEIDR model captures the entire epidemic, detected and not detected. Prior to observing RSV cases, the epidemic cycle started within the undetected population. This offset parameter was estimated as a constant parameter across years and also allowed to vary by epidemic year.

### Model fitting

The nonlinear equations were solved using the lsoda function from the odesolve library [18] in R statistical software [19]. The parameters were estimated using a grid search. Two fitting statistics were used. The estimates were the values that minimized the square root of the sum of standardized squared errors (RSE) and/or the square root of the sum of squared standardized errors (RMSE). The RSE was calculated as the square root of the sum of the squared errors between the observed daily cases and the fitted model, divided by the fitted value, , where  was the fitted value on day *i*. The RMSE was calculated as the square root of the sum of the squared weighted errors between the observed daily cases and the fitted model; the weight being the fitted value, . The denominator from these measures adjusted for the magnitude of the epidemic curve to avoid fitting the model mainly to the peak, where differences could over-inflate the fitting statistic and under-value differences during the early and late stages of the epidemic. The RMSE reduces the effect of fit to the peak more than does the RSE.

A grid search was used starting with an initial wide range of values for f, β, and α. The search grid was repeated with successively narrowing ranges to minimize the RSE. The grid started with the range of reasonable values, 0 - 1 for β and f and one to 200 days for α. The range was reduced and resolution increased iteratively around minimal RSE and RMSE values. The minimum grid resolution was 0.0001 for β, 0.01 for f, and one day for α. The RSEs and RMSEs from the grid search results were used to select the best parameter estimates within each model type (eg, one model type had only transmission rates that varied by epidemic year).

The model with all three parameters allowed to vary by epidemic year was fit as a saturated model to provide a benchmark for RSE and RMSE, along with the Schwarz Criteria described below, and percent error in estimating epidemic size when evaluating more parsimonious models in which only one of the 3 parameters was allowed to vary by epidemic year. Multiple measures were used to compare the models, in part because the Schwarz criteria assumed the residuals were independent and identically distributed, which was not the case; they are, in fact, autocorrelated.

The Schwarz Information Criterion [20] were calculated based on the weighted least squares method used for parameter estimation. There were *n *= 2555 data points, 365 days of case data for each of seven years, and *k*, the number of parameters estimated was 28 in the full model (four parameters for seven years) and 16 in each other model (two parameters for seven years and two parameters overall). The Schwarz Criteria were calculated as:  where M represents either the RSE or RMSE fit statistic [21]. The absolute values of the percent error in estimating total epidemic size were summed across seasons for comparison of models.

## Results

### Descriptive Analysis

The number of children with test-positive RSV infection ranged from 682 cases in 2004-5 to 1704 cases in 2007-8 (Table 1). The median size of the annual epidemic was 1113 cases. Overall, 98% of cases were detected between the months of October and April. Larger epidemics alternated with smaller epidemics. The amplitude of this biennial cycle was approximately 600 cases.

**Table 1:** Observed RSV epidemic size, start date, days to peak, duration, and 4-week exponential growth.

Years (Epidemic Year)	Epidemic Size	Observed Start (t_0_)	Days Until Peak	Duration	Exponential Growth
2001-2 (1)	1074	12/12/01	69	124	0.063

2002-3 (2)	733	12/4/02	83	175	0.034

2003-4 (3)	1553	11/19/03	75	142	0.081

2004-5 (4)	682	11/27/04	85	173	0.033

2005-6 (5)	1400	11/02/05	62	154	0.068

2006-7 (6)	1113	11/12/06	80	176	0.061

2007-8 (7)	1704	11/27/07	59	144	0.050

The total number of children (under 18 years of age) tested per epidemic year ranged from approximately 3000 to 7000, with numbers of tests increasing over time. Overall, 21% percent of these were positive for RSV, varying according to the biennial cycle. Of children tested, 81% were less than three years old and 95% were less than 11 years old. Of children with positive tests, 92% were less than three years old and 99% were less than 11 years old. Of the children tested, 70% were from Salt Lake County and 77% of children with positive tests were from Salt Lake County.

### Regression analyses

Exponential growth rates calculated from cases accumulated for four weeks from the observed epidemic season start ranged from 0.034 to 0.081 (Table 1) across the epidemic seasons. The effective reproductive numbers ranged from 1.27 to 1.49 using a serial interval of seven days [16]. In regression analyses (Table 2), the four-week exponential growth rate exhibited a substantial positive correlation with epidemic size (r = 0.69, p = 0.08), and was negatively correlated with start day (r = -0.43, p-value = 0.33), days to peak (r = -0.44, p-value = 0.32), and length of the epidemic (r = -0.58, p-value = 0.17). The regression models provided estimates of epidemic season characteristics that were on average within 16% of observed epidemic season size, 11% of observed days to peak, and 8% of observed epidemic length. Using exponential growth rates calculated from weeks one through six provided, in general, increasing correlation (Table 3).

**Table 2:** Results of regression analysis using exponential growth to predict epidemic size, days to peak, and length.

	Total Epidemic Size	Days to Epidemic Season Peak	Length of Epidemic Season
Regression Intercept (S.E.)	321.0 (417)	87.5 (13.5)	192.3 (24.0)

Regression Slope (S.E.)	15383 (7175)	-255.3 (233)	-659 (413)

Regression Model p-value	0.08	0.32	0.17

R^2^	0.48	0.19	0.20

Root Mean Square Error	17.6	3.2	4.2

Mean of Absolute % Error	16	11	8

**Table 3:** Correlations between exponential growth rate (calculated at weeks one through six) with observed RSV epidemic size, start date, days to peak, and duration.

Epidemic Weeks used for Exponential Growth Rate	Epidemic Size	Observed Start (t_0_)	Days Until Peak	Duration
1	0.30	-0.52	0.17	0.09

2	0.58	-0.63	-0.33	-0.30

3	0.62	-0.51	-0.36	-0.47

4	0.69	-0.43	-0.44	-0.58

5	0.78	-0.40	-0.56	-0.64

6	0.81	-0,40	-0.59	-0.62

### SEIDR model

The saturated SEIDR model was fit to seven epidemic years of observed RSV data with epidemic year-specific RSE values that ranged from 13 to 21, RMSE values that ranged from 0.40 to 0.77 and percent error of total cases that ranged from 1% to 16%. The fit statistics for the models with either transmission parameter or detection fraction estimated as a constant across epidemic year did not differ substantially from those from the saturated model (Table 4). The minimum RSE model with detection fraction held constant across epidemic years had the smallest % error, smallest Schwarz RSE Criterion, and had other fit statistics nearly equal to the saturated model. The minimum RMSE models were, in general, fitting to the tails of the epidemic and resulted in large errors in estimating epidemic size.

**Table 4:** Fit statistics for models with different sets of parameters allowed to vary across epidemic year.

Model:	Min RSE Models		Min RMSE Models		Schwarz Criterion	
	
Parameters that vary by epidemic year	Sum RSE	Sum % Error	Sum RMSE	Sum % Error	RSE	RMSE
Time Offset	128	114	4.3	197	20050	2905

Transmission Parameter	152	127	4.6	217	20992	3288

Detection Fraction	154	202	4.6	221	21073	3287

Time Offset & Transmission Parameter	115	58	3.8	179	19556	2239

Time Offset & Detection Fraction	122	110	4.1	185	19871	2736

Transmission Parameter & Detection Fraction	147	119	4.4	218	20936	3254

All	113	75	3.5	188	19578	1946

The RSE and RMSE column values represent the models with the minimum of the fit statistic over that category. The % error is the sum of the absolute values of the % error in estimating epidemic size.

**Figure 3:**
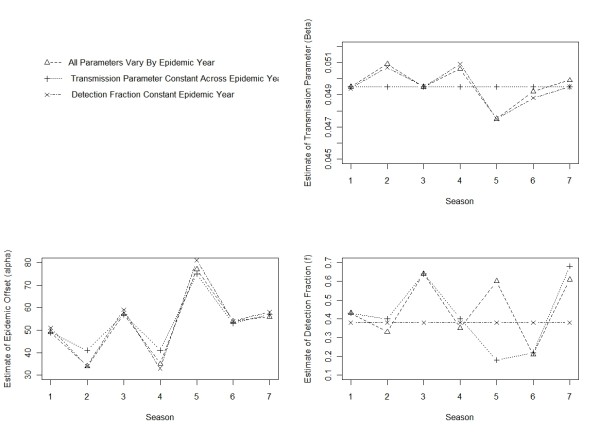
**SEIDR model parameter estimates**. SEIDR model parameter estimates for three models for each of the 7 seasons. The parameters are transmission parameter (top right), epidemic offset (bottom left), and detection fraction (bottom right). The estimates from the model with 1) all parameters varying by epidemic year are open triangles, 2) transmission parameter constant across epidemic year are plus signs, and 3) detection fraction constant across epidemic year are x's. The data were collected by Primary Children's Medical Center, Salt Lake City, UT from July 2001 through June 2008.

The pattern of variation in estimates of offset from all models matched the biennial cycle variation in total epidemic size across epidemic years (Figure 3). The variation in estimates of the transmission parameter and detection fraction did not necessarily match this cycle for all epidemic years. The parameter estimates for the transmission parameter were negatively correlated with total epidemic size.

## Discussion

The SEIDR model we presented made assumptions that simplified the reality of RSV transmission. We have identified three limitations to the SEIDR modeling effort. First, the population age separation does not take full advantage of differences in interaction among a non-homogenous population. Second, related to this, the parameter values were not allowed to vary within the population. Transmission, for instance, could be age-dependent (due, eg, to hand-washing habits). Third, the grid search method of parameter estimation did not provide estimated standard errors for parameter estimates, which limited the ability to compare models and seasons.

Despite these limitations, this SEIDR model was useful; it modeled the observed RSV cases from PCMC as part of larger unobserved epidemic seasons and provided a framework for investigating the model parameters. The parameters offset and transmission may not be completely identifiable within this framework but more likely represent combined other forces unmeasured here.

Our future work includes addressing these limitations and expanding the complexity of the models. RSV is carried by all age groups but is, in general, only a concern for infants. Thus, an age-stratified model, possibly with different mixing mechanisms, would more closely resemble the true transmission. The biennial cycle of large, early, and short seasonal epidemics followed by smaller, later, and longer seasonal epidemics the next year observed in Utah is similar to other published studies of seasonal RSV epidemics in temperate climates. The theories for this phenomenon include the existence and switching of two RSV disease strains, climate patterns, and waning immunity after infection [6,8,9,22-24]. These and other theories could be investigated in more complex models. It is understood that immunity after infection of RSV is partial, at best. This incomplete immunity and severity of re-infections could be incorporated into more complex models [8,25]. Finally, future modeling efforts will involve approaches that include measures of uncertainty in parameter estimates, including Bayesian methods [26,27] and likelihood and other methods [28,29].

## Conclusions

The first main conclusion of this work was that exponential growth was somewhat empirically related to seasonal epidemic characteristics. The variations in epidemic seasons from data collected at PCMC during the seven years of the study can be partially explained by the variation in exponential growth, especially characteristics of epidemic size, peak day, and length of the epidemic. The seven years of data were not sufficient to make conclusive statements on the nature of the relationships. These early findings based on just seven data points can be built upon to explore early prediction of the upcoming RSV epidemic season. These early predictions could be used by hospitals to budget and allocate resources and to coordinate the timing of palivizumab treatment. They can be used by public health to advise clinicians and the public and also to help identify non-standard epidemics earlier in the season. For example, health departments might take specific actions if the number of observed cases during the season greatly exceeds early predictions.

**Figure 4:**
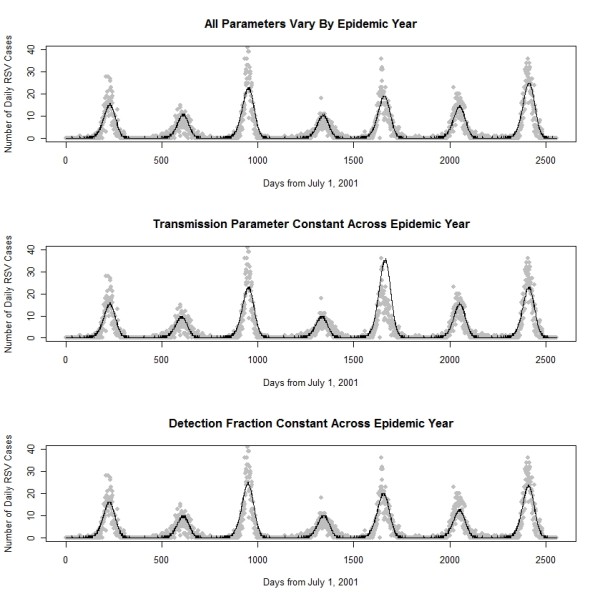
**Observed RSV cases and model predicted epidemic curves**. Observed RSV cases (grey dots) collected by Primary Children's Medical Center in Salt Lake City from July 2001 through June 2008, plotted for each season along with fitted SEIDR models.

The second main conclusion of this work was that variation of the transmission parameter and the start of the epidemic (offset) over epidemic years could explain the variation in seasonal epidemic size. The three model parameters allowed to vary by epidemic year (detection fraction, transmission parameter, and offset) provided possible rationale for the variation in seasonal epidemic size. The model with detection fraction held constant across epidemic year fits the observed data well with the fewest parameters. The parameter estimates from this model also match the expected biennial pattern of the epidemic years. From the models considered in this study, this one performs best overall (Figure 4).

## Competing interests

The authors declare that they have no competing interests.

## Authors' contributions

ML performed the analysis and wrote the bulk of the manuscript. PG helped to conceive the study and prepare the data and also wrote a large part of the Introduction, Methods, and Discussion sections of the text. TG advised on the design of the study's analysis and helped prepare the Methods and Results sections of the text. NW acquired and managed the data. AG provided clinical insight and helped conduct the literature review. RR helped write the Introduction and Discussion sections of the text, providing public health perspective to the study. CB helped conduct the literature review and write the Introduction and Discussion sections of the text. MS conceived the study and directed its implementation, including contributions to all sections of the text. All authors read and approved the final manuscript.

## Pre-publication history

The pre-publication history for this paper can be accessed here:

http://www.biomedcentral.com/1471-2334/11/105/prepub

## References

[B1] Stensballe L Devasundaram J Simoes E Respiratory syncytial virus epidemics: the ups and downs of a seasonal virus Pediatr Infect Dis J 2003 22 2 Suppl S21 32 12671449 10.1097/01.inf.0000053882.70365.c9

[B2] Forster J Ihorst G Rieger C Stephan V Frank H Gurth H Berner R Rohwedder A Werchau H Schumacher M Tsai T Petersen G Prospective population-based study of viral lower respiratory tract infections in children under 3 years of age (the PRIDE study) Eur J Pediatr 2004 163 12 709 716 10.1007/s00431-004-1523-9 15372233

[B3] Leader S Kohlhase K Recent trends in severe respiratory syncytial virus (RSV) among US infants, 1997 to 2000 J Pediatr 2003 143 5 Suppl S127 132 14615711 10.1067/s0022-3476(03)00510-9

[B4] Paramore L Ciuryla V Ciesla G Liu L Economic impact of respiratory syncytial virus-related illness in the US: an analysis of national databases Pharmacoeconomics 2004 22 5 275 284 10.2165/00019053-200422050-00001 15061677

[B5] Shay D Holman R Newman R Liu L Stout J Anderson L Bronchiolitis-associated hospitalizations among US children, 1980-1996 JAMA 1999 282 15 1440 1446 10.1001/jama.282.15.1440 10535434

[B6] Terletskaia-Ladwig E Enders G Schalasta G Enders M Defining the timing of respiratory syncytial virus (RSV) outbreaks: an epidemiological study BMC Infect Dis 2005 5 1 20. 10.1186/1471-2334-5-20 15801975 PMC1084247

[B7] Panozzo C Fowlkes A Anderson L Variation in timing of respiratory syncytial virus outbreaks: lessons from national surveillance Pediatr Infect Dis J 2007 26 11 Suppl S41 45 18090199 10.1097/INF.0b013e318157da82

[B8] Weber A Weber M Milligan P Modeling epidemics caused by respiratory syncytial virus (RSV) Math Biosci 2001 172 2 95 113 10.1016/S0025-5564(01)00066-9 11520501

[B9] White L Mandl J Gomes M Bodley-Tickell A Cane P Perez-Brena P Aguilar J Siqueira M Portes S Straliotto S Waris M Nokes D Medley G Understanding the transmission dynamics of respiratory syncytial virus using multiple time series and nested models Mathematical Biosciences 2007 209 222 239 10.1016/j.mbs.2006.08.018 17335858 PMC3724053

[B10] Lipsitch M Cohen T Cooper B Robins J Ma S James L Gopalakrishna G Chew S Tan C Samore M Fisman D Murray M Transmission dynamics and control of severe acute respiratory syndrome Science 2003 300 1966 1970 10.1126/science.1086616 12766207 PMC2760158

[B11] Lipsitch M Bergstrom C Invited commentary: real-time tracking of control measures for emerging infections Am J Epidemiol 2004 160 6 517 519 discussion 520 10.1093/aje/kwh256 15353410

[B12] Wallinga J Teunis P Different epidemic curves for severe acute respiratory syndrome reveal similar impacts of control measures Am J Epidemiol 2004 160 6 509 516 10.1093/aje/kwh255 15353409 PMC7110200

[B13] U.S. Census Bureau Population Division-Counties http://www.census.gov/popest/counties/CO-EST2008-01.html

[B14] U.S. Census Bureau Population Division-States http://www.census.gov/popest/states/NST-ann-est.html

[B15] Koopman J Modeling infection transmission Annu Rev Public Health 2004 25 303 326 10.1146/annurev.publhealth.25.102802.124353 15015922

[B16] Crowcroft N Zambon M Harrison T Mok Q Heath P Miller E Respiratory syncytial virus infection in infants admitted to paediatric intensive care units in London, and in their families Eur J Pediatr 2008 167 4 395 399 10.1007/s00431-007-0509-9 17541638

[B17] Hall C Douglas R Geiman J Respiratory syncytial virus infections in infants: quantitation and duration of shedding J Pediatr 1976 89 1 11 15 10.1016/S0022-3476(76)80918-3 180274

[B18] Setzer R odesolve: Solvers for Ordinary Differential Equations R package version 0.5-18 edn 2007 Vienna, Austria: R Foundation for Statistical Computing

[B19] R Development Core Team A Language and Environment for Statistical Computing 2007 Vienna, Austria: R Foundation for Statistical Computing

[B20] Cavanaugh J Neath A Generalizing the derivation of the Schwarz information criterion Communications in Statistics - Theory and Methods 1999 28 1 49 66 10.1080/03610929908832282

[B21] Landaw E DiStefano J Multiexponential, multicompartmental and non-compartmental modeling, II: Data analysis and statistical considerations American Journal of Physiology (Regulatory Integrative Comparative Physiology 15) 1984 246 665 677 10.1152/ajpregu.1984.246.5.R665 6720989

[B22] Waris M Pattern of respiratory syncytial virus epidemics in Finland: two-year cycles with alternating prevalence of groups A and B Journal of Infectious Diseases 1991 163 3 464 10.1093/infdis/163.3.464 1995719

[B23] Hall C Walsh E Schnabel K Long C McConnochie K Hildreth S Anderson L Occurrence of groups A and B of respiratory syncytial virus over 15 years: associated epidemiologic and clinical characteristics in hospitalized and ambulatory children Journal of Infectious Diseases 1990 162 6 1283 10.1093/infdis/162.6.1283 2230258

[B24] Dietz K L S The incidence of infectious diseases under the influence of seasonal fluctuation Lecture Notes in Biomathematics 1976 11 New York: Springer

[B25] Novotni D Weber A A stochastic method for solving inverse problems in epidemic modeling Proceedings of the International Conference on Mathematics and Engineering Techniques in Medicine and Biological Sciences 2003 467 473

[B26] O'Neill P Roberts G Bayesian inference for partially observed stochastic epidemics Journal of the Royal Statistical Society 1999 162 1 121 129 10.1111/1467-985X.00125

[B27] Glass K Becker N Clements M Predicting case numbers during infectious disease outbreaks when some cases are undiagnosed Statistics in Medicine 2007 26 171 183 10.1002/sim.2523 16479555

[B28] Ionides E Breto C King A Inference for nonlinear dynamical systems PNAS 2006 103 49 18438 18443 10.1073/pnas.0603181103 17121996 PMC3020138

[B29] Becker N Mollison D Statistical challenges of epidemic data Epidemic Models: Their structure and relation to data 1995 Cambridge: Cambridge University Press 339 349

